# Global transcriptional profiling reveals *Streptococcus agalactiae *genes controlled by the MtaR transcription factor

**DOI:** 10.1186/1471-2164-9-607

**Published:** 2008-12-16

**Authors:** Joshua D Bryan, Roxanne Liles, Urska Cvek, Marjan Trutschl, Daniel Shelver

**Affiliations:** 1 Department of Microbiology and Immunology, Louisiana State University Health Sciences Center in Shreveport, Shreveport, Louisiana 71130, USA; 2 Department of Computer Science, Louisiana State University in Shreveport, Shreveport, Louisiana 71115, USA; 3 Center for Molecular and Tumor Virology, Louisiana State University Health Sciences Center in Shreveport, Shreveport, Louisiana 71130, USA

## Abstract

**Background:**

*Streptococcus agalactiae *(group B *Streptococcus*; GBS) is a significant bacterial pathogen of neonates and an emerging pathogen of adults. Though transcriptional regulators are abundantly encoded on the GBS genome, their role in GBS pathogenesis is poorly understood. The *mtaR *gene encodes a putative LysR-type transcriptional regulator that is critical for the full virulence of GBS. Previous studies have shown that an *mtaR*^- ^mutant transports methionine at reduced rates and grows poorly in normal human plasma not supplemented with methionine. The decreased virulence of the *mtaR *mutant was correlated with a methionine transport defect; however, no MtaR-regulated genes were identified.

**Results:**

Microarray analysis of wild-type GBS and an *mtaR *mutant revealed differential expression of 12 genes, including 1 upregulated and 11 downregulated genes in the *mtaR *mutant. Among the downregulated genes, we identified a cluster of cotranscribed genes encoding a putative methionine transporter (*metQ1NP*) and peptidase (*pdsM*). The expression of four genes potentially involved in arginine transport (*artPQ*) and arginine biosynthesis (*argGH*) was downregulated and these genes localized to two transcriptional units. The virulence factor *cspA*, which encodes an extracellular protease, was downregulated. Additionally, the SAN_1255 locus, which putatively encodes a protein displaying similarity to plasminogen activators, was downregulated.

**Conclusion:**

To our knowledge, this is the first study to describe the global influence of MtaR on GBS gene expression. This study implicates the *metQ1NP *genes as encoding the MtaR-regulated methionine transporter, which may provide a mechanistic explanation for the methionine-dependent growth defect of the *mtaR *mutant. In addition to modulating the expression of genes involved in metabolism and amino acid transport, inactivation of *mtaR *affected the expression of other GBS genes implicated in pathogenesis. These findings suggest the possibility that MtaR may play a multifaceted role in GBS pathogenesis by regulating the expression of numerous genes.

## Background

*Streptococcus agalactiae *(group B *Streptococcus*; GBS) is a Gram-positive bacterial pathogen of humans. GBS is best known as a pathogen of neonates, in which it is a leading cause of pneumonia, sepsis, and meningitis. In recent years, GBS has also emerged as a significant pathogen of the immunocompromised, elderly, and adults with underlying medical conditions [1].

GBS infections progress rapidly, in part due to the vigorous growth of the organism *in vivo*. Classical virulence factors such as toxins have been the predominant focus of most virulence studies; however, recent studies reflect increasing interest in the connection between bacterial growth, metabolism, and virulence [2-5]. The metabolic processes of bacteria used during *in vivo *growth or survival are prerequisites for virulence that have often been overlooked in pathogenesis studies [5], with the notable exception of iron utilization. Similar to iron, other nutrients may be scarce *in vivo *and may require significant metabolic adaptation for *in vivo *survival. Because the host environment is dynamic and ill-defined in terms of available nutrients, the metabolic processes of pathogenic bacteria that are critical for *in vivo *survival are not easily predictable without experimentation. Exploration of this understudied facet of bacterial pathogenesis may lead to novel therapies directed against bacterial metabolism [2]. Interestingly, most currently-available antibiotics target essential fundamental bacterial processes including metabolism.

In neonatal infections, GBS has the striking ability to rapidly transition through a diverse range of host environments and exhibits vigorous growth in many of these settings, despite its very limited biosynthetic capacities [6]. In early-onset neonatal disease, GBS first colonizes the maternal vagina, ascends the birth canal, penetrates the placental membranes, and proliferates rapidly within the amniotic fluid. The fetus may aspirate the infected amniotic fluid during parturition, which can lead to neonatal pneumonia. GBS can then penetrate and damage cellular barriers, transcytose through cells, and enter the bloodstream. From the bloodstream, the bacterium can disseminate to multiple organs and penetrate the blood-brain barrier, leading to meningitis [7].

The mechanisms underlying the ability of GBS to thrive in diverse host environments are largely uncharacterized. However, the numerous regulatory proteins encoded within the GBS genome may reflect the adaptability of the bacterium. The spectrum of genes controlled by GBS transcription factors will undoubtedly provide insight into how GBS adapts to the human host. However, compared with other pathogens such as *Streptococcus pyogenes*, only a few global transcriptional profiling studies have been conducted to examine the function of regulatory proteins in GBS [8-10].

The GBS *mtaR *gene displays homology to LysR-type transcription regulators (LTTR) and is required for GBS virulence in a neonatal rat model of sepsis [11]. An insertional inactivation of *mtaR *resulted in a mutant displaying approximately a 1000-fold increase in LD_50_. Furthermore, coinfection experiments have shown that the *mtaR *mutant survives very poorly *in vivo*, as compared with the wild-type strain [11].

During the course of an infection, GBS often invades the bloodstream and induces a dangerous systemic inflammatory response that can lead to fatal sepsis. Experiments using human plasma (as an ex-*vivo *model for the nutritional conditions present in the bloodstream) revealed that inactivation of *mtaR *causes a methionine-dependent growth defect in GBS. Furthermore, it was also shown that the *mtaR *mutant transported methionine poorly. Thus, it was hypothesized that MtaR controls a gene or gene(s) involved in methionine transport and/or metabolism [11]. However, no direct experimental evidence supporting this hypothesis has been reported. Interestingly, methionine is one of the least abundant amino acids in normal human blood; thus, methionine scavenging may be a critical determinant for the survival of GBS as well as other fastidious pathogens in *vivo*.

In this study, we performed transcriptional profiling analyses to identify MtaR-regulated genes involved in methionine transport, methionine metabolism, and other processes related to GBS virulence. The findings of our study implicate a single gene cluster as encoding the MtaR-controlled methionine transport system. No other genes predicted to be involved in methionine metabolism or transport were found to be affected by the *mtaR *mutation. Expression of genes implicated in a variety of other processes including systemic virulence, interaction with fibrinogen, metabolism, and transport of other amino acids were affected by the mutation of *mtaR*. Our findings reveal that MtaR influences expression of a range of GBS genes and suggest that MtaR may play a multifactorial role in GBS virulence.

## Results

### Establishment of bacterial growth conditions and microarray analysis

**Figure 1:**
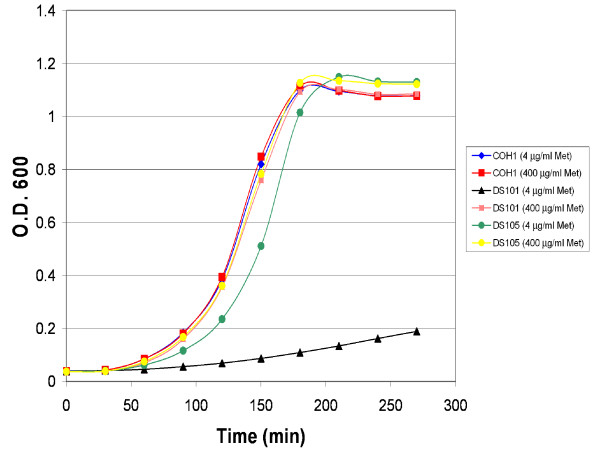
**Growth of GBS strains COH1, DS101 (*mtaR*^-^), and DS105 (*mtaR*^+/-^) in chemically defined medium (CDM)**. GBS strains were cultured statically at 37°C in CDM with either 4 μg/ml or 400 μg/ml methionine. Samples were withdrawn every thirty minutes and optical density was measured at 600 nm (O.D._600_). Cells were collected at O.D._600 _= 0.3 for harvest of RNA.

The GBS strain COH1 [12], a clinical isolate from a case of fatal infant septicaemia belonging to the hypervirulent ST-17 lineage [13] was the reference strain for this study. An isogenic GBS *mtaR *mutant (DS101) bearing a kanamycin-resistance cassette insertion was derived by precise allelic exchange and was utilized as the test strain. The DS101 strain was previously shown to exhibit a methionine transport defect when grown in chemically defined medium (CDM) at high methionine concentrations [11]. Mutation of GBS *mtaR *significantly attenuated growth at low methionine levels (4 μg/ml), while the wild-type strain grows normally in the presence of 4 μg/ml methionine (Fig. 1). As nutritional starvation can induce mRNA degradation and growth rate can modulate gene expression, we cultured the wild-type and *mtaR *mutant strains under conditions in which the strains exhibited identical growth kinetics (in media containing 400 μg/ml methionine) (Fig. 1).

In preparation for microarray experiments, three cultures each of COH1 (wild type) and DS101 (*mtaR*::*kan*) were grown to an OD_600 _of 0.3 in CDM containing 400 μg/ml methionine. RNA was harvested and processed for hybridization to microarrays as described above in Materials and Methods. A custom oligonucleotide microarray (Affymetrix, Santa Clara, CA) based on the GBS COH1 genomic sequence [14] was utilized for microarray analysis. The entire procedure was repeated three times, giving three biological replicates for each strain.

Genes were considered differentially expressed if the following three criteria were met: 1) There was a two-fold or greater difference in signal intensity for the probe set corresponding to the gene, 2) statistical analysis (see Materials and Methods and Additional File 1) yielded a P value less than 0.001, and 3) real-time PCR (qPCR) validated that the gene was differentially regulated. Genes were divided into functional categories using a combination of published experimental evidence and homology searches, and the twelve genes meeting the above criteria are presented in Table 1. Upon review of the list of differentially-expressed genes, each gene that was identified as differentially regulated according to the above criteria was found to have been assigned "Present" calls by the Affymetrix GCOS software for both strains in all three replicate experiments. The microarray results correlated strongly with the qPCR results (Table 2), indicating the validity of our data.

**Table 1:** *S. agalactiae *genes that are differentially regulated in the *mtaR *mutant.

Locus (gene)	Function/annotation/homology	P value	Fold change
*Transport and binding proteins*			
SAN_1753 (*metP*)	ABC transporter, permease	4.4 × 10^-6^	-2.43
SAN_1754 (*metN*)	ABC transporter, ATP-binding	5.8 × 10^-7^	-2.43
SAN_1756 (*metQ*)	ABC transporter, substrate-binding	3.5 × 10^-11^	-5.30
SAN_0595 (*artP*)	ABC transporter, permease	2.5 × 10^-7^	-2.35
SAN_0596 (*artQ*)	ABC transporter, ATP-binding	9.7 × 10^-6^	-2.44
*Protein fate: degradation of proteins, peptides, and glycopeptides*			
SAN_1755 (*pdsM*)	Peptidase, M20/M25/M40 family	2.1 × 10^-9^	-3.72
SAN_2186 (*cspA*)	Serine protease	2.3 × 10^-7^	-3.20
SAN_1255	Streptokinase-like	1.4 × 10^-5^	-2.65
*Amino acid biosynthesis*			
SAN_0156 (*argG*)	Argininosuccinate synthase	8.6 × 10^-5^	-2.60
SAN_0157 (*argH*)	Argininosuccinate lyase	8.7 × 10^-6^	-2.91
*Energy metabolism*			
SAN_0597 (*manB*)	Phosphomannomutase or Phosphoglucomutase	4.1 × 10^-5^	-2.04
*Hypothetical conserved*			
SAN_0933		8.3 × 10^-4^	+2.47

The differentially-regulated genes are grouped into functional categories, based on experimental evidence, protein database searches, and genome annotation. Each GBS COH1 gene [14] is indicated by its Comprehensive Microbial Resources locus number (SAN number) available on the J. Craig Venter Institute website  and gene symbol, when applicable. Fold changes in gene expression (*mtaR *mutant relative to wild-type) and Bayesian P values were derived using Cyber-T [38] analysis of data from three independent biological replicates. Criteria for inclusion in this table were the following: Bayesian P value < 0.001, greater than two-fold change, and confirmation of differential expression by qPCR (Table 2).

**Table 2:** Validation of differential gene expression observed in microarray experiments via qPCR.

Gene	mRNA level change		Sense primer sequence (5'-3')	Antisense primer sequence (5'-3')
			
	qRT-PCR	microarray		
SAN_1753	-2.1	-2.4	GGGTTGGGAAGGTGCTTAC	TAAACCTCCAATAAGGAACGAC
SAN_1754	-3.0	-2.4	ATGGAATTGTCGGTTATTCAGGAG	GTCAGTGTCACCTTGTTGTCG
SAN_1756	-6.5	-5.3	GCTCCAATTCGTATCTATTCTG	ATTTGATTAAACCTGCTGACTG
SAN_0595	-3.7	-2.4	AAGATAGTGCTCTCCTTCAAAC	CCCTTTCCCAAGATATTTCTCC
SAN_0596	-1.6	-2.4	GCAGGGATTATTGTTGAGC	GAAGTCTCTTGTGCGGATTTC
SAN_1755	-6.0	-3.7	GCAGATGAGGTAGAACAGTGG	GTGCGGTAATGTGACCTTTATC
SAN_2186	-3.7	-3.2	AATATAAGTTAGGTGCCGTATCTG	CGTGTTGTTAGTAGGTGTCTC
SAN_1255	-3.2	-2.7	AAACCCAAATCCTCACATTATTG	TCCCATCTTTACATTGACTTCG
SAN_0156	-1.7	-2.6	ATATAAAGGTTCTGCCAAAGTC	TGTATAAGTTGCTAAGTTCTCATC
SAN_0157	-2.8	-2.9	TTGGACATCATCTAATGGCTTAC	ATATGGCGGTCAATAGGAAATG
SAN_0597	-2.1	-2.0	GGCGGGATTGAGAGGTAAAC	TAACGGACATCATAACTAACTGC
SAN_0933	+1.7	+2.5	CAGAAATAAGGCATCAACTACC	AAAGAATAGGCATTATCAATACG

### Complementation analysis

The *mtaR *mutant bears a kanamycin cassette insertion in the 5' region of the *mtaR *derived by precise allelic replacement of the wild-type *mtaR *allele [11]. The genes flanking *mtaR *on both sides are transcribed in the opposite direction [11], indicating the mutation is not polar. Previous complementation analysis, performed by introducing the wild-type *mtaR *allele into the mutant in *trans *[11], confirmed that the *mtaR *mutation was not polar and was responsible for the attenuation of virulence and the methionine-dependent growth defect observed in plasma. Introduction of the wild-type *mtaR *allele in *trans *also restored the growth of the *mtaR *mutant in 4 μg/ml CDM (Fig. 1), indicating that the *mtaR *mutation was also responsible for the methionine-dependent growth defect in CDM.

Complementation analysis was performed on six representative genes to confirm that the differential regulation resulted from the engineered *mtaR *mutation and not from an unlikely spurious secondary mutation. We measured gene expression by qPCR in the *mtaR *mutant (DS101) and the *mtaR *mutant in which the wild-type *mtaR *allele has been introduced in *trans *(DS105). For each strain, the results are expressed as a ratio of gene expression of the test strain to that of the wild-type strain: *metQ1*, 0.16 (DS101/COH1), 0.99 (DS105/COH1); *cspA*, 0.30 (DS101/COH1), 0.98 (DS105/COH1); *artP*, 0.27 (DS101/COH1), 1.23 (DS105/COH1); *artQ*, 0.63 (DS101/COH1), 1.07 (DS105/COH1); SAN_1255, 0.5 (DS101/COH1), 0.93 (DS105/COH1). A gene, *metQ2*, that was not differentially expressed in the mutant served as a control, and its expression level (in relation to the wild-type strain) was not increased when the wild-type *mtaR *allele was introduced to the *mtaR *mutant: *metQ2*, 1.15 (DS101/COH1), 1.02 (DS105/COH1). These data indicate that in DS105 (DS101 with the wild-type *mtaR *allele in *trans*), expression of the differentially-expressed genes was restored to approximately wild-type levels. Taken together, the complementation results strongly suggest that the *mtaR *mutation was responsible for the differential gene regulation observed in this study.

### Methionine transport genes

**Figure 2:**
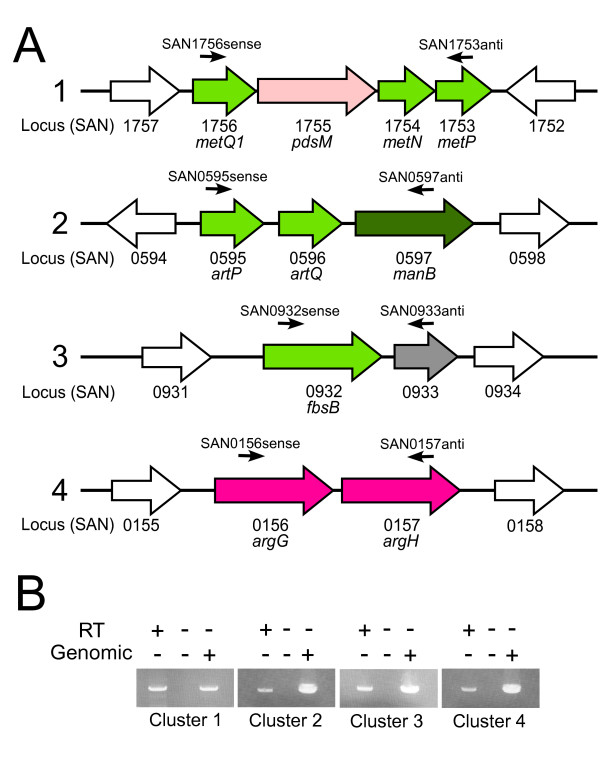
**Gene clusters under the control of MtaR**. (A) Genes differentially expressed in the *mtaR *mutant that cluster in four regions of the GBS COH1 chromosome. The filled, colored arrows represent genes differentially expressed in the *mtaR *mutant. The color of the arrow corresponds to the functional category predicted or established for the each gene: light green, transport and binding; light pink, protein fate; dark green, energy metabolism; grey, hypothetical, and dark pink, amino acid biosynthesis. Open arrows represent genes not differentially expressed in the *mtaR *mutant. The arrows above the gene clusters represent primers used for transcriptional linkage experiments depicted in panel B. (B) RT-PCR was performed using RNA harvested from GBS strain COH1. PCR reactions were performed with or without RT enzyme or chromosomal DNA. The number of the cluster analyzed in each experiment is indicated above the corresponding gel. The designation and location of the primers utilized are indicated in panel A and the primer sequences are listed in the text.

The expression of a gene cluster (*metQ1NP*) (Fig. 2A) that displayed strong similarity to established methionine transport gene clusters in other Gram-positive bacteria was downregulated in the *mtaR *mutant. RT-PCR analysis revealed that the gene cluster was cotranscribed (Fig. 2B). This cluster is predicted to encode products that are highly similar to components of bacterial ABC transporters. The first component of typical bacterial ABC transporters consists of an ATP-binding cassette (ABC), which binds and hydrolyzes ATP. The second component is a permease, which forms a channel in the membrane. The third component, a substrate-binding protein, imparts specificity to the system. We identified genes predicted to encode the ABC component (MetN), the permease component (MetP), and the substrate-binding component (MetQ1) in the cluster and found that these genes were downregulated in the *mtaR *mutant. A gene in this cluster (*pdsM*) is predicted to encode a peptidase from the M20/M25/M40 family; the cotranscription of this gene in the *metQ1 *cluster may suggest the encoded protein is involved in the breakdown of peptides for nutritional purposes.

**Figure 3:**
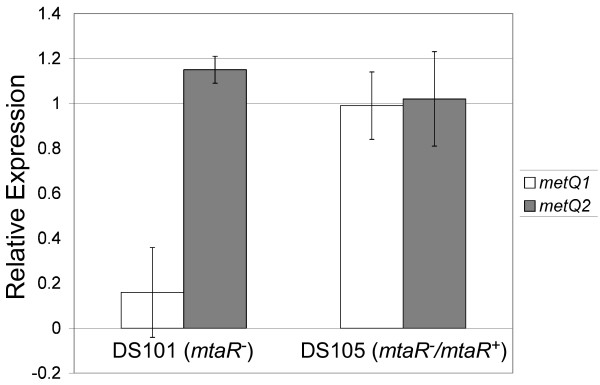
**qPCR evaluation of genes encoding putative methionine transport components**. qPCR was used to examine MtaR-dependent expression of putative methionine ABC transport genes (substrate-binding components). Melting curve analysis and agarose electrophoresis of PCR products were performed to ensure that each primer pair only produced a single amplicon. The ΔΔC_t _quantification method was used to determine relative gene expression differences normalized to *rpsL *(housekeeping control) expression. *metQ1 *expression (open bars) and *metQ2 *expression (filled bars) were compared between strains. The comparisons were performed by calculating the gene expression ratios of DS101 (*mtaR*^-^) or DS105 (DS101 complemented with a low-copy plasmid bearing the wild-type *mtaR*^+ ^allele) to those of COH1.

In a previous bioinformatics study, a putative gene (*metQ2*) located elsewhere on the chromosome was predicated to encode a methionine substrate-binding protein; this gene was hypothesized to be regulated by MtaR [15,16]. In our study, microarray analysis indicated that the *metQ2 *gene is expressed (data not shown). However, qPCR analysis revealed no statistically-significant difference in *metQ2 *expression between the *mtaR *mutant and the wild-type strain (Fig. 3).

### Genes involved in arginine and carbohydrate metabolism

A putative operon (*artPQmanB*) that is predicted to encode components of another ABC transport system and a potential phosphomannomutase/phosphoglucomutase was also downregulated in the *mtaR *mutant. The GBS *artPQ *genes exhibit strongest similarity to arginine and glutamine transporters. The *artPQ *gene products are unlikely to be involved in glutamine transport since the GBS glutamine transporter has been identified [17]. The *manB *gene, located adjacent to *artQ *(Fig. 2A), is predicted to encode a protein with similarity to phosphomannomutases and phosphoglucomutases. The *artPQ *and *manB *genes were shown to be cotranscribed via RT-PCR analysis (Fig. 2B).

Two additional genes potentially involved in arginine metabolism, *argG *and *argH*, were downregulated in the *mtaR *mutant. SAN_0156 (*argG*) is predicted to encode argininosuccinate synthase. The predicted product of SAN_0157 (*argH*) displayed homology to argininosuccinate lyase, which catalyzes the formation of arginine from argininosuccinate. In other bacteria, ArgG and ArgH catalyze the two terminal reactions in the biosynthesis of arginine. The *argG *and *argH *genes are clustered (Fig. 2A) and the results of our RT-PCR analysis (Fig. 2B) indicate these genes are cotranscribed.

### Genes encoding products involved in the GBS-fibrinogen interaction

Two genes (*cspA *and *fbsB*) that encode products involved in the interaction between GBS and fibrinogen were differentially expressed in the *mtaR *mutant. Expression of the virulence-associated *cspA *gene [18], which encodes a member of the surface-localized, extracellular cell-envelope proteases (CEPs) [19], was downregulated. The *cspA *gene is necessary for virulence and cleavage of fibrinogen by GBS [18]. Heterologously-expressed CspA has been shown to cleave fibrinogen [20]. In addition, the expression of *fsbB *(SAN_0932), which encodes a fibrinogen-binding protein, was upregulated by approximately 1.82 fold by microarray analysis (P = .0035; PPDE [<P] = 0.92). While slightly below the criteria for inclusion in Table 1, qPCR confirmed that *fbsB *was upregulated by 1.75 fold in the *mtaR *mutant. Moreover, a gene of unknown function that is adjacent to *fbsB *(SAN_0933) was upregulated by ~2.5-fold (Table 1) in the *mtaR *mutant. The results of our RT-PCR analysis (Fig. 2B) indicate that *fbsB *and SAN_0933 are cotranscribed. Taken together, these findings indicate that *fbsB *expression is under the control of MtaR.

## Discussion

Our findings strongly suggest that the *metQ1NP *genes encode the MtaR-regulated methionine transport system. Additionally, we found that the expression of four genes potentially related to arginine metabolism (*artPQ *and *argGH*) were under the influence of MtaR. Furthermore, a gene (*manB*) possibly involved in carbohydrate metabolism was found to be controlled by MtaR. Of note, carbohydrate metabolism has recently been linked to the pathogenesis of a number of streptococci [3,21]. In addition to modulating expression of genes involved in transport and metabolic processes, our study also indicates MtaR influences the expression of genes (*fbsB *and *cspA*) involved in other aspects of GBS pathogenesis. Overall, 12 genes met the criteria for being differentially regulated. Given the level of replication (3 biological replicates per strain), there remains the possibility of false negatives; it is possible that some genes in the MtaR-dependent regulon, including those with less than a two-fold change in expression, have not been revealed in this study.

### Methionine transport

The *metQ1NP *gene cluster was the only putative methionine transport system affected by the *mtaR *mutation. Inactivation of *metQ1NP *homologs in *Bacillus subtilis *[22] and *Streptococcus mutans *[23] abolished methionine transport. Our preliminary attempts to inactivate the *metQ1 *gene (via plasmid insertion-duplication mutagenesis) were unsuccessful and appeared to arrest growth of the organism (J. Bryan and D. Shelver, unpublished results), even when the medium was supplemented with high (400 μg/ml) concentrations of methionine or peptide methionine sources. The simplest interpretation of these results is that the cluster encodes a sole GBS methionine transporter. However, because the mutation we attempted to engineer is predicted to be polar onto downstream genes, it is uncertain which of the components are essential for growth. Future studies are necessary to test the hypothesis that *metQ1NP *encodes the sole GBS methionine transport system.

### MtaR controls genes encoding products associated with GBS-fibrinogen interaction

In addition to regulating amino acid transport and metabolism, MtaR also appears to control the expression of genes involved in the interaction between GBS and fibrinogen. Fibrinogen is a host protein that plays a key role in many facets of bacterial pathogenesis and is a mediator of the host defense against pathogens [24,25]. However, pathogenic bacteria can subvert fibrinogen and the associated coagulation cascade for their own purposes [25]. For example, fibrinogen can be used by pathogenic bacteria for adherence [26] and evasion of the innate immune system [27].

Expression of least two genes involved or implicated in the interaction between GBS and fibrinogen were differentially expressed in the *mtaR *mutant. In particular, the expression of *fbsB *was upregulated. In addition to binding fibrinogen, the encoded product (FbsB) promotes GBS invasion of epithelial cells [28]. Additionally, the *cspA *gene, which encodes a serine protease from the cell-envelope family of proteases (CEPs) [19], was downregulated in the *mtaR *mutant. All reports of CEPs in pathogenic streptococci indicate that these proteins play roles in virulence [18,19,29,30]. CspA has the ability to promote cleavage of the Aα subunit of fibrinogen at a single site [18]. However, it cleaves very few other substrates (see paragraph below), indicating a high level of specificity. The *cspA *gene is not necessary for GBS growth in plasma or laboratory broth (THB, CDM) and COH1 cells do not cleave the general protease substrate casein regardless of the presence or absence of a functional *cspA *gene [18], (T. O. Harris and C. E. Rubens, personal communication). Thus, similar to the CEPs of other pathogenic streptococci, the GBS CspA protein does not appear to play a nutritional role [18].

The *cspA *gene is required for the full virulence of GBS in a rat model of neonatal sepsis [18] but the connection between the fibrinogen-cleaving activity of CspA and GBS virulence is not yet clear. However, previous studies have shown that CspA contributes to neutrophil evasion by promoting resistance to opsonophagocytic killing [18]. Furthermore, CspA proteolyzes certain members of the CXC class of chemokines, abolishing their ability to attract neutrophils (J. Bryan and D. Shelver, unpublished data). Thus, in addition to promoting GBS survival *in vivo *by regulating the *metQ1NP *cluster, MtaR may contribute to GBS virulence by upregulating the expression of CspA.

Numerous recent studies have focused on a *Streptococcus pyogenes *CEP (SpyCEP/ScpC) that is also capable of proteolyzing CXC chemokines [30-33]. This protease has been implicated in necrotizing fasciitis and contributes to the virulence of *S. pyogenes *in an animal model of spreading necrosis. Similar to CspA of GBS, *S. pyogenes *SpyCEP/ScpC is thought to be a virulence factor that allows *S. pyogenes *to evade neutrophils. However, regulation of *spyCEP *by an MtaR homolog has not been reported.

Our results revealed that a gene of unknown function (SAN_1255), whose product shares similarity with the plasminogen activators of a variety of bacteria (i. e., 20% identity and 35% similarity to *Streptococcus uberis *PauB [34]), was found to be downregulated. Plasminogen is the inactive form of a human protease that, when converted to a highly active protease (plasmin), cleaves fibrin (the polymerized form of fibrinogen found in clots) as well as a number of other host proteins. Bacterial plasminogen activators are potent virulence factors that promote invasive infections and bacterial spread [24]. However, it has not been determined if GBS can activate plasminogen in the absence of host factors [35]; thus, the function of SAN_1255 remains unclear. Due to the observation that SAN_1255 exhibits considerable strain-to-strain allelic variability, it has been speculated that its product may interact with the host [36].

### The MET box and MtaR-dependent regulation of gene expression

In a previous bioinformatics study, Rodionov et al. observed that a region of two-fold rotational symmetry (the MET box) precedes two GBS genes (*metQ1 *and *metQ2*) predicted to encode products similar to bacterial methionine transport components and a gene (*metE*) whose predicted product shows homology with enzymes that catalyze the conversion of homocysteine to methionine [15]. The MET box is characterized by the TATAGTTTnAAACTATA consensus sequence [16], and strongly resembles a LysR-type transcriptional regulatory protein binding site.

During the course of our studies, a report was published describing an apparent *S. mutans *MtaR ortholog, MetR, that is 80% identical to MtaR [23]. This study identified MET boxes upstream of several genes encoding components of the methionine biosynthetic pathway as well as a cluster encoding a methionine transporter [23]. In electrophoretic mobility shift assays, purified *S. mutans *MetR bound to DNA fragments containing MET boxes; however, DNA footprinting was not performed to specifically locate the MetR binding site. The authors speculated that streptococcal MtaR/MetR homologs act specifically as regulators of methionine metabolism and activate gene expression in response to methionine starvation. However, this study did not globally examine genes influenced by MetR (i.e., genes not preceded by MET boxes). Some genes preceded by MET boxes were not subject to regulation by MetR and methionine starvation. Also, MetR bound to some DNA fragments containing MET boxes but did not affect regulation of these genes. The correlation between MET boxes and MetR regulation was thus incomplete. It is therefore not clear if MET boxes are the sole determinants of MetR-dependent regulation.

In our study, despite the presence of MET boxes preceding three genes potentially related to methionine transport and metabolism (*metQ1*, *metQ2*, and *metE*), only expression of the *metQ1NP *transport cluster was affected by the *mtaR *mutation. Furthermore, computerized searches of DNA regions in the vicinity of other MtaR-regulated genes did not reveal MET boxes. These results can be explained if MtaR has the ability to regulate genes by binding to sequences other than MET boxes or if MtaR influences expression of the differentially-regulated genes indirectly via other regulatory proteins. Localization of MtaR binding sites by DNAse footprinting or other techniques has not yet been possible since MtaR, like a number of other LTTRs, has been recalcitrant to expression in an active form. However, efforts to purify MtaR and definitively identify its binding site are ongoing. Regardless of whether MtaR influences expression of genes in a direct or indirect manner, our findings provide insight into the ability of MtaR to regulate genes involved in a variety of processes in GBS.

## Conclusion

To our knowledge, this is the first study to describe the spectrum of genes controlled by MtaR. Our findings indicate that MtaR activates expression of a specific methionine transport gene cluster (*metQ1NP*), which may allow the organism to efficiently scavenge methionine *in vivo*. Genes potentially involved in other aspects of metabolism (e.g., arginine transport and sugar metabolism) were also identified as influenced by MtaR. In addition, our findings reveal that MtaR controls the expression of two previously-characterized genes (*fbsB *and *cspA*) that have been implicated in other facets of GBS pathogenesis.

## Methods

### Materials (chemicals, media, and molecular biology reagents)

Media for the routine growth of *Escherichia col*i (LB broth) and *Streptococcus agalactiae *(Todd-Hewitt Broth; THB) were purchased from Becton, Dickinson and Company (Sparks, MD). Chemically-defined media (CDM) was prepared as described by Willett and Morse [37] with the exception that methionine was added to the final concentrations indicated. Chemical reagents were purchased from Sigma-Aldrich (St. Louis, MO) unless otherwise noted.

### Strains and plasmids

*Streptococcus agalactiae *strain COH1 is a minimally-passaged clinical isolate obtained from a fatal case of human infant septicaemia [12] and belongs to hypervirulent ST-17 lineage [13]. The DS101 strain (*mtaR*^-^) is an isogenic derivative of COH1 that harbors a kanamycin-resistance cassette insertion in *mtaR *[11]. Plasmid pDS8, utilized for complementation of DS101 [11], is a low-copy plasmid bearing *mtaR*. The DS105 strain is an isogenic derivative of DS101 that harbors pDS8 [11].

### DNA microarray design

A custom DNA oligonucleotide microarray (LSUHSC-S_Shelver_GBS_5K_V1.0) was designed by Affymetrix (Santa Clara, CA) and manufactured by Nimblegen (Madison, WI) for use on the Affymetrix GeneChip^® ^platform. The NimbleExpress™ 49 format with a 17 micron feature size was utilized. Oligonucleotides (25-mers) were selected according to the genomic sequence of GBS strain COH1 [14]. Each potential coding region was represented by 24 perfectly matched (PM) and 24 mismatched (MM) oligonucleotides when possible. The oligonucleotide sets were tiled twice on each chip. The microarray design has been deposited in the Gene Expression Omnibus (GEO)  under accession number GPL7515.

### RNA isolation

In preparation for by real-time PCR analysis (qPCR), GBS strains were cultured at 37°C in CDM to an O.D._600 _of 0.3 and 50 ml of cells were harvested by centrifugation. The GBS cells were then placed in a 950 μl suspension containing QBio lysing matrix B, 50 mM sodium acetate pH 4.0, 0.5% SDS, and 25% acidic phenol (Amresco; Solon, OH). Cells were mechanically disrupted with the lysing matrix using a FastPrep^® ^120 device (MP Biomedicals; Solon, OH) with a speed setting of 5 and a pulse length of 22 seconds. The mixture was separated by centrifugation for 10 minutes. The aqueous phase of the suspension was extracted twice with equal volumes of acidic phenol/chloroform (Amresco), followed by an equal volume of chloroform. RNA was then precipitated by adding two volumes of 100% ethanol and sodium acetate pH 5.2 to a final concentration 0.3 M. Samples were incubating at -20°C for 1 hour, pelleted in a refrigerated centrifuge, dried, and resuspended in diethylpyrocarbonate (DEPC)-treated water. Residual DNA was then removed via treatment with RNase-free DNaseI (Promega; Madison, WI) and purification with an RNeasy (Qiagen; Valencia, CA) column according to the manufacturer's instructions. RNA was stored at -80°C.

In preparation for microarray analysis, the protocol described above was modified as follows. After cell disruption and recovery of the aqueous phase, RNA was precipitated via the addition of two volumes of 100% ethanol and sodium acetate (pH 5.2) to a final concentration of 0.3 M. TRIzol reagent (1 ml; Sigma-Aldrich) was then added and the mixture was incubated at room temperature for 5 minutes Chloroform (200 μl) was added, shaken vigorously, and allowed to remain at room temperature for 2 minutes. The mixture was then centrifuged for 10 minutes, the aqueous phase was recovered and RNA was precipitated with an equal volume of 100% isopropanol. The pellet was air-dried and resuspended in 50 μl of DEPC-treated water. Residual DNA was removed as described above.

### Real-time PCR (qPCR) analysis

Appropriate primers were designed using the Beacon Design program (Premier Biosoft International, Palo Alto, CA). The purified RNA was then used as template in the Superscript III first strand synthesis kit (Invitrogen; Carlsbad, CA). The resulting cDNA was then used as template in a PCR reaction with SYBR Green and iTaq (Bio-Rad; Hercules, CA). Fluorescence of the SYBR Green was measured using a Bio-Rad iCycler thermocycler. Melting curve analyses and electrophoresis of PCR products on agarose gels were performed to ensure that each primer pair only produced a single amplicon. The ΔΔC_T _method was used to estimate fold changes in gene expression between the COH1 and DS101 strains using *rpsL *(a housekeeping gene) as a reference. To ensure the ΔΔC_T _method was valid for estimating relative transcript levels, primer validation was performed to demonstrate that the efficiencies of target and reference were approximately equal and that the absolute values of the slopes of log input RNA amount versus ΔC_T _were < 0.1.

### Microarray analysis

The integrity of the purified RNA was assessed using an Agilent 2100 Bioanalyzer and a RNA 6000 Nano LabChip Kit (Agilent; Palo Alto, CA). GeneChip targets were prepared according to the manufacturer's protocols (Prokaryotic Expression Manual, Affymetrix). The cDNA samples were synthesized from 10 μg of total RNA using Superscript II reverse transcriptase (Invitrogen). Carryover RNA was degraded using 1 N NaOH followed by treatment with 1 N HCl. The cDNA samples were purified and concentrated using a MinElute™ PCR Purification kit (Qiagen). Samples were fragmented using DNase I (Promega; Madison, WI) and biotin end-labeled using the GeneChip DNA Labeling Reagent (Affymetrix). A hybridization cocktail containing 3.9 μg labeled cDNA was hybridized to the arrays. Hybridization was performed for 16 hours with a rotation of 60 rpm at a temperature of 45°C in a GeneChip Hybridization Oven 640 (Affymetrix). Arrays were washed and stained with a streptavidin-phycoerythrin conjugate using predefined protocols with a GeneChip Fluidics Station 450 (protocol ProkGE-WS2_450). Arrays were scanned with a GeneChip Scanner 300 7G (Affymetrix). Scanned microarray images were analyzed using GeneChip Operating Software 1.4 (Affymetrix). Arrays were globally scaled to a target intensity value of 500 to compare individual experiments. The microarray data has been deposited in the Gene Expression Omnibus (GEO)  under accession number GSE13325.

Genes were assessed for differential expression using Cyber-T [38] version 8.01  with the following settings: the Bayesian prior estimate was 10, the sliding window size was 101, and the β-fit iteration value was 2. We defined the cutoff value for differential gene expression as transcripts that showed a >2.0-fold change and a Bayesian t-test P value of < 0.001. The posterior probability of differential expression (PPDE) (< p) value for the genes identified was > 0.964. The results of the Cyber-T analysis for all genes are included as Additional file 1.

### RT-PCR transcriptional mapping

RT-PCR was used to test if differentially-regulated genes present in clusters (Fig. 2) were cotranscribed. RNA was isolated as described above for qPCR analysis. Reverse transcription was accomplished by combining 2 pmol of sequence-specific primers, 5'TAAACCTCCAATAAGGAACGAC3' (SAN1753anti), 5'CAAAGAATAGGCATTATCAATACG3' (SAN0933anti), 5'ATAACGGACATCATAACTAACTGC3' (SAN0597anti), 5'ATATGGCGGTCAATAGGAAATG3' (SAN0157anti) with 250 ng of RNA and dNTPs were added to a final concentration of 1 mM. The resulting mixture was heated to 70°C for 5 minutes and placed immediately on ice. RNasin (Promega), DTT, and M-MLV reverse transcriptase were then added. This mixture was incubated for 10 minutes at 25°C, 50 minutes at 37°C, and 15 minutes at 70°C. The cDNA was then digested with RNaseH (Invitrogen) to remove RNA/DNA hybrids. For the subsequent PCR reactions, the following primer pairs were used: SAN1756sense (5'GCTCCAATTCGTATCTATTCTG3') and SAN1753anti; SAN0595sense (5'AAGATAGTGCTCTCCTTCAAAC3') and SAN0597anti; SAN0932sense (5'AACAGTTAATCAGTATGAAGCG3') and SAN0933anti; and SAN0156sense (5'CTGTCTGTATGGATGTTGGC3') and SAN0157anti. The primer locations within the clusters are indicated on Fig. 2A. GBS chromosomal DNA was included in control reactions. PCR products were visualized by agarose gel electrophoresis.

### Gene identification and similarity (TIGR/BLAST)

Gene identification was performed by BLAST searches [39], ClustalW alignments [40], and annotation of GBS and related genomes . Searches for MET boxes or sequences similar to MET boxes were conducted as described [41].

## Authors' contributions

JB assisted with the design of experiments, conducted the microarray and PCR experiments, and assisted in drafting the manuscript. RL assisted in the RT-PCR transcriptional mapping experiments. UC and MT assisted in the analysis of the microarray data, the MET box analysis, and the drafting of the manuscript. DS conceived of the study, assisted with the design of experiments, coordinated the experiments and drafted and edited the manuscript. All authors read and approved the final manuscript.

## Supplementary Material

Additional file 1 **Analysis of gene expression data using Cyber-T**. Click here for file
